# Conversion of Soluble Compounds in Distillery Wastewater into Fungal Biomass and Metabolites Using Australian *Ganoderma* Isolates

**DOI:** 10.3390/jof11060432

**Published:** 2025-06-06

**Authors:** Aline D. O. Campos, Hashini J. Wahalathanthrige, Shane Russell, Mark D. Harrison, Peter James Strong

**Affiliations:** 1 Centre for Agriculture and the Bioeconomy, Queensland University of Technology, Brisbane 4000, Australia; aline.deoliveiracampos@hdr.qut.edu.au (A.D.O.C.); hashini.wahalathanthrige@hdr.qut.edu.au (H.J.W.); md.harrison@qut.edu.au (M.D.H.); 2 School of Biology and Environmental Science, Queensland University of Technology, Brisbane 4000, Australia; 3 Central Analytical Research Facility, Queensland University of Technology, Brisbane 4000, Australia; sc.russell@qut.edu.au; 4 School of Mechanical, Medical and Process Engineering, Queensland University of Technology, Brisbane 4000, Australia

**Keywords:** white-rot fungi, submerged fermentation, stillage, β-glucan, waste valorization, biodegradation

## Abstract

Stillage is an acidic residue from ethanol production that has a high carbon load. Here, *Ganoderma* isolates were assessed for the treatment of rum stillage while producing biomass and associated metabolites. Isolates grew in 25% raw stillage, removing up to 73% of soluble organic carbon, 77% soluble nitrogen, and 74% phenolic compounds. Isolate G2 demonstrated faster removal of organic carbon and nitrogen. Biomass and metabolite production were benchmarked against a nutrient medium. In stillage, maximum values of the following were obtained: 8.2 g·L^−1^ biomass; 52.8% crude protein; 22.1 mg·g^−1^ extractable protein; antioxidants of 17.2 mg TE·g^−1^ (2,2′-azino-di-(3-ethylbenzothiazoline-6-sulfonic acid), ABTS) and 16.6 µmol Fe^2+^·g^−1^ (ferric reducing antioxidant power, FRAP); 2.9 mg GAE·g^−1^ phenolic compounds (gallic acid equivalents); 1.2% lipids; and 11% β-glucans. In the nutrient medium, the following were obtained: 6.9 g·L^−1^ biomass; 56.4% crude protein; 38.7 mg·g^−1^ extractable protein; antioxidants of 24.9 mg TE·g^−1^ (ABTS) and 25.9 µmol Fe^2+^·g^−1^ (FRAP); 6.0 mg GAE·g^−1^ phenolic compounds; 0.7% lipids; and 13% β-glucans. To our knowledge, this is the first report detailing the biomass metabolite content of *Ganoderma* mycelium using rum stillage. The production of edible biomass containing bioactive products demonstrates the potential of using *Ganoderma* strains to valorize this residue.

## 1. Introduction

Distillery wastewater, or stillage, is a by-product of the agro-processing industries that presents a significant global challenge due to its polluting characteristics [1]. During ethanol production, sugars from various agricultural stocks are fermented anaerobically, and the resultant ethanol is removed by distillation [1]. The distillery industry ranks as the second-largest generator of industrial wastewater globally [1]. In Brazil, for example, each liter of ethanol generates approximately 12 L of stillage, culminating in 360 billion liters of wastewater annually [2]. This residue typically has an acidic pH, a dark color, and a high chemical oxygen demand (COD up to 150 g·L^−1^) and biological oxygen demand (BOD up to 50 g·L^−1^), which can deplete oxygen levels and impact aquatic life [1]. It also contains inhibitory compounds, such as phenolics, melanoidins, and other recalcitrant substances [1]. Global volumes are likely to increase, especially if ethanol production for sustainable aviation fuels becomes a reality.

Using stillage as a fungal growth substrate offers a low-cost, dual-purpose strategy—pollutant reduction and biomass valorization [3]. White-rot fungi secrete extracellular enzymes capable of mineralizing lignin that are able to degrade a broad range of recalcitrant organic compounds, including dyes, phenolics, and pharmaceuticals [3]. These compounds can be metabolized into proteins, β-glucans, enzymes, and other metabolites with applications in the food, feed, or fuel sectors [3].

While fungal bioremediation offers a promising alternative to conventional methods, it is not without challenges. The slow growth rate of some filamentous fungus increases the risk of contamination by faster-growing organisms, such as bacteria, yeast, and mold fungi, hindering culture stability [3,4,5]. Fungal and bacterial growth depends on controlled pH and nutrient supply, which can raise process costs [6]. Lastly, some fungal species may not be suitable for all applications, requiring careful selection [3]. However, fungi can be more effective than bacteria at degrading lignin-like compounds. Thermotolerant strains, such as *Daedaleopsis* species and *Ganoderma* strains, have achieved over 90% COD and ammonia reduction and can convert oil-rich waste into biomass [7]. Fungal biomass is easier to recover, filter, and dry than bacterial biomass, and serves as a food or feed protein. The parallel production of fungal biomass during the bioremediation process is advantageous due to their metabolic versatility and the value of fungal biomass, which can potentially offset treatment costs [3].

The recovered fungal mycelium from stillage presents a significant opportunity for valorization due to its diverse bioactive and structural components. Its protein content makes it a viable ingredient for animal feed, contributing to a more sustainable livestock production [6]. The structure and composition of fungal mycelium makes it suitable for use in biomaterials, such as myco-leather or biocomposites [8]. The presence of bioactive molecules, like β-glucans, provides avenues for nutraceuticals and functional food ingredients, given their immunomodulatory and health-enhancing properties [7]. This multipurpose biomass underscores the economic and environmental benefits of integrating fungal biomass recovery into wastewater treatment systems.

To reduce production costs, low-value substrates are being explored as alternatives to conventional media. Several studies report the successful cultivation of *Ganoderma* spp. in industrial effluents or alternative media [6,7,9,10,11,12]. For instance, *G. lucidum* has grown well in thin stillage from rice spirit supplemented with glucose or molasses, with adjustments to pH supporting mycelial biomass and polysaccharide yields [11,12]. Similar outcomes have been achieved using cheese whey, sugarcane, wine, and olive mill wastewater [6,9,10]. *Ganoderma* has also shown high removal efficiency for phenolics, color, and BOD in textile and olive mill effluents [10]. While their cultivation on various agro-industrial by-products has been documented, limited data exists regarding their use in rum distillery wastewater, particularly in relation to the concurrent evaluation of proteins, β-glucans, antioxidant compounds, and lipids.

This study evaluated Australian *Ganoderma* strains for their capacity to generate biomass and reduce organic carbon, nitrogen, and phenolics loads in rum stillage as a low-cost substrate. Fungal growth, metabolite yields, and reductions in key wastewater parameters were quantified in submerged cultures using 25% stillage as a growth medium. The influence of culture conditions on biomass accumulation and metabolite profiles was investigated by time course analysis. Performance in stillage was benchmarked against a nutrient medium. The results demonstrate the potential of *Ganoderma*-based systems for integrated biomass valorization and the treatment of distillery wastewater.

## 2. Materials and Methods

### 2.1. Materials

Dimethyl sulfoxide (DMSO), methanol, n-hexane, sulfuric acid, vanillin reagent, ABTS (2,2-azino-bis(3-ethylbenzothiazoline-6-sulphonic acid)), agar, BSA, ammonium sulphate, Anthrone, ergosterol, gallic acid, glucose, malt extract, potassium phosphate monobasic, TPTZ (2,4,6-Tris(2-pyridyl)-s-triazine), Trolox, urea, and yeast extract were purchased from Sigma-Aldrich (St. Louis, MO, USA). Peptone and glacial acetic acid were purchased from Thermo Fisher Scientific (Waltham, MA, USA). Ferric chloride, Folin–Ciocalteu’s reagent, hydrochloric acid, magnesium sulphate, phosphoric acid 85% (*w*/*w*), sodium carbonate, and sodium hydroxide were purchased from ChemSupply (Adelaide, SA, Australia). The β-glucan assay kit (yeast and mushroom) was purchased from Megazyme (Bray, Ireland). Bradford reagent was obtained from Bio-Rad (Hercules, CA, USA).

### 2.2. Ganoderma Cultivation and Inoculum Preparation

*Ganoderma* strains were isolated from live fruiting bodies (Queensland and New South Wales, Australia), obtained between 2019 and 2023. Macroscopic characteristics of each fruiting body are described in Table 1. An in-depth macroscopic and microscopic morphological description of these fruiting bodies is described elsewhere [13]. Mycelial stock cultures were prepared in 10% (*v*/*v*) DMSO and stored at −80 °C. Cryogenic stocks were resuscitated on plates containing agar (13 g·L^−1^); yeast, malt extract, and peptone (5 g·L^−1^); glucose (20 g·L^−1^); and magnesium sulphate and potassium phosphate monobasic (0.5 g·L^−1^) at 28 °C for 120 h. Mycelial plugs were transferred into a nutrient medium (same composition, without agar) at 25 °C for 120 h. Biomass was transferred to the nutrient medium containing 2.5% stillage to acclimate the culture to wastewater. Actively growing mycelia were washed and used in subsequent experiments.

### 2.3. Collection and Characterization of Distillery Wastewater

Stillage was collected on 16th March 2024 from the Bundaberg Rum Distillery (Queensland, Australia) in two 10 L high-density polyethylene containers and stored at 4 °C until use. The stillage characteristics, including pH, soluble organic carbon (SOC), total nitrogen (TN), and total phenolic content (TPC) are described in Table 2.

### 2.4. Culture Media Optimization

#### 2.4.1. Wastewater Concentration and pH

Seven *Ganoderma* isolates were assessed for their growth in different concentrations of stillage. Pre-acclimated cultures were blended, washed, and resuspended in distilled water (1:10 *v*/*v*) into 24 deep-well plates at final concentrations of 15, 20, 25, and 30% (*v*/*v*) stillage. In a pre-assessment, 40% stillage inhibited growth (Supplementary Figure S1). Abiotic and water-only controls were included. Plates were incubated at room temperature for 10 days at 150 rpm. Fungal growth was estimated by measuring dry biomass yield. Four isolates were selected based on growth and inoculated into 24 deep-well plates containing the selected stillage concentration (25%) at different initial pH values (original pH of 4.8, 5.5, 6.0, and 6.5). The same incubation conditions and controls were applied. Biomass yield and pH variations were measured, and the most promising strains were selected for further fermentation and metabolite assessment.

#### 2.4.2. Nitrogen Supplementation

The three most promising *Ganoderma* strains were evaluated for their response to nitrogen supplementation in stillage. Conical flasks (250 mL) containing 120 mL of 25% stillage at the original pH were supplemented with yeast extract, ammonium sulphate, or urea at the same nitrogen-equivalent concentrations in yeast extract:

- 0.5 and 2 g·L^−1^ of yeast extract;
- 0.11 g·L^−1^ and 0.43 g·L^−1^ of urea; and
- 0.24 g·L^−1^ and 0.94 g·L^−1^ of ammonium sulphate.

To facilitate data analysis, the different nitrogen concentrations are displayed as 0.5 and 2 g·L^−1^ nitrogen equivalent of yeast extract.

Pre-acclimated mycelia were washed and resuspended in 25% stillage before being inoculated (1:10 *v*/*v*) in conical flasks. Flasks were incubated at room temperature, shaking at 150 rpm, for up to 16 days. Abiotic controls and water-only cultures were included. Samples were centrifuged (3720 g for 5 min, and 21,380 g for 5 min) and supernatant analyzed for soluble organic carbon and total phenolic compounds. Samples were measured in triplicate, unless otherwise specified.

### 2.5. Metabolite Production in Stillage Versus Nutrient Medium

Metabolite production and bioremediation potential of *Ganoderma* isolates were evaluated in 25% stillage and nutrient medium (control). The control medium contained 5 g·L^−1^ of yeast, malt extract, and peptone, 20 g·L^−1^ glucose, and 0.5 g·L^−1^ magnesium sulphate and potassium phosphate monobasic. Prior to inoculation, isolates were acclimated in nutrient media containing 20% (*v*/*v*) stillage at 25 °C for three days. Mycelia were centrifuged, washed, divided in half, and resuspended equally in either 25% stillage or a nutrient medium. The inoculum was added to media (120 mL) and incubated at 25 °C, shaking at 150 rpm, for up to 12 days, with samples removed at 4-day internals. The resulting mycelial biomass was washed with distilled water to remove the residual medium, freeze-dried (VirTis Benchtop Pro, SP Scientific, Warminster, PA, USA), and stored at −20 °C. Analyses included the quantitation of β-glucans, crude protein, soluble proteins, total lipids, total phenolics, and antioxidant activity (ABTS and FRAP assays). For stillage-based cultures, soluble organic carbon (SOC), total phenolics, and soluble nitrogen (SN) were monitored to assess the bioremediation capacity of each isolate.

#### 2.5.1. Glucan Quantification

Total, α-, and β-glucan contents of *Ganoderma* mycelial samples and their respective fruiting bodies were quantified spectrophotometrically using a yeast and mushroom β-glucan assay kit following the manufacturer’s instructions (Megazyme, Wicklow, Ireland). β-glucan content was calculated as the difference between total and α-glucan content. Freeze-dried biomass was pulverized using a TissueLyser II cell disruptor (QIAGEN, Hilden, Germany) and used for glucan analysis. In short, total glucans were extracted with 12 M sulfuric acid, neutralized, and enzymatically digested with exo-1,3-β-glucanase (20 U·mL^−1^) and β-glucosidase (4 U·mL^−1^) to determine total glucans and free glucose. For α-glucan quantification, dried mycelia were extracted with 1.7 M NaOH and digested with amyloglucosidase (1630 U·mL^−1^) and invertase (500 U·mL^−1^). In both assays, supernatants were incubated with GOPOD reagent, and absorbance was measured at 510 nm. Yeast powder containing ~50% β-glucans was used as a control.

#### 2.5.2. Aqueous and Hexane Extraction

Freeze-dried fungal mycelial biomass (50 mg) was extracted in a mix of 0.5 mL of n-hexane and 1 mL of deionized water in a Sonicator at 60 °C for 30 min with frequent shaking. The aqueous and hexane layers were separated by centrifugation using a benchtop microcentrifuge (#5425 R G, Eppendorf, Germany) at 21,380 g for 5 min. The formed layers were filtered through a 0.22 µm polypropylene syringe filter and aliquoted to a clean microtube. The n-hexane portion was used for the total lipids assay, while the aqueous extract was assessed for total proteins, total polysaccharides, total phenolics, and antioxidants.

#### 2.5.3. Crude and Soluble Proteins

Total nitrogen was measured in 2 mg of dried mycelium using the FlashSMART Elemental Analyzer (Thermo Scientific, MA, USA), and crude protein was then estimated by multiplying the total nitrogen content by 6.25. Soluble proteins were detected spectrophotometrically at 595 nm according to the Bradford assay [14]. A calibration curve of bovine serum albumin (BSA) was used as the standard (1 to 0.05 g·L^−1^).

#### 2.5.4. Antioxidant Activity of Ganoderma Extracts

##### ABTS Radical-Scavenging Activity

The ABTS radical-scavenging activity was assessed using a modified method based on the ABTS assay [15]. A stock solution was prepared by mixing 2.45 mM ammonium persulfate with 7.40 mM ABTS solution. This mixture was incubated in the dark at room temperature for 16 h to allow radical formation. A fresh working solution was prepared by diluting the stock solution at 1:20 in water to achieve an absorbance of 1.00 ± 0.05 at 734 nm. For the assay, 300 µL of the ABTS working solution was combined with 5 µL of aqueous *Ganoderma* mycelial extract. The reaction mixture was incubated in the dark at room temperature for 60 min, after which the absorbance was measured at 734 nm using a microplate reader (BioTek Synergy HTX Multimode Reader, Agilent, Santa Clara, CA, USA). Extracts were appropriately diluted to ensure measurements fell within the linear range of the Trolox calibration curve (0.025–2 mM). The results are expressed as millimoles of Trolox equivalents per gram of dry biomass.

##### Ferric Reducing Antioxidant Ability (FRAP)

A FRAP working solution was prepared daily by mixing 0.3 M acetate buffer at pH 3.6 with 10 mM 2,4,6-Tris(2-pyridyl)-s-triazine (TPTZ) in 40 mM hydrochloric acid and 20 mM ferric chloride at a ratio of 10:1:1 [16]. This mixture was warmed in a water bath at 37 °C until completely dissolved. To measure FRAP activity, 15 µL of aqueous *Ganoderma* extract was combined with 285 µL of the warm FRAP working solution and incubated at 37 °C in the dark. Absorbance was measured at 593 nm after 6 min. Extracts were diluted as necessary to match the linear response range of the ferric chloride (0.1–2 mM), and the results are expressed as millimoles of ferric chloride per gram of dry biomass.

##### Total Phenolic Compounds

The total phenolic content (TPC) of wastewater supernatants, and mycelial aqueous extracts was determined following Folin–Ciocalteu’s method. Aliquots of clarified samples were appropriately diluted with MilliQ water before analysis, and 20 µL of the sample was added to 160 µL of water in flat-bottom 96-well plates. A total of 20 µL of Folin–Ciocalteu’s reagent was added and incubated at room temperature for 5 min. Then, 100 µL of sodium carbonate (75 g·L^−1^) was added and the plate was incubated in the dark at room temperature for 60 min. Gallic acid (79–150 mg·L^−1^) was used as the standard, and the results are expressed as milligrams of gallic acid equivalent per gram of dry biomass (mg GAE·g^−1^).

#### 2.5.5. Total Lipids

Total lipid content was quantified using the sulfo-phospho-vanillin method with minor modifications [17]. Hexane extracts (50 uL) were evaporated to dryness at 50 °C, and the resulting powder was resuspended in 1 mL of sulfuric acid, briefly vortexed, and heated at 90 °C for 10 min. The reaction was cooled to room temperature and 2 mL of phospho-vanillin reagent was added. Phospho-vanillin reagent was prepared daily by mixing 120 mg of vanillin with 20 mL of hot water and 80 mL of phosphoric acid at 85% (*v*/*v*). The reaction was heated in a water bath at 37 °C for 15 min. Finally, the absorbance was measured at 530 nm against the reagent blank. A calibration curve of ergosterol in sulfuric acid (0.2 to 2.0 g·L^−1^) was used to estimate the lipid content in the samples.

#### 2.5.6. Soluble Organic Carbon and Soluble Nitrogen

The SOC and SN in the stillage medium were measured before and after fermentation. Stillage samples were diluted 500× in water and 50 mL of diluted material was analyzed using TOC-L with a TNM attachment (Shimadzu Corporation, Kyoto, Japan).

#### 2.5.7. Statistical Analysis

Statistical analysis was conducted in GraphPad Prism (v 10.4.2). All measurements were conducted in triplicate (*n* = 3) unless otherwise specified, and the data represents a mean ± standard deviation. Two-way ANOVA was used to assess the effect of media, time, and their interaction. When significant effects were observed (*p* < 0.05), a multiple comparison test was used for post hoc analysis with the Bonferroni correction (α = 0.05). Due to the large number of pairwise comparisons, it was not feasible to display all statistically significant differences within the figures. Instead, we highlighted either the earliest time point at which a significant variation was observed, the comparison with the strongest significance, and/or the contrast of metabolite yields between media to illustrate key trends. In addition, Pearson correlation (*p* < 0.05, *n* = 4) was performed to explore the relationships between biomass production, antioxidant activity, metabolite yields, and stillage remediation. Pearson correlation coefficients and corresponding *p*-values are provided in the Supplementary Materials (Tables S1–S6).

## 3. Results and Discussion

### 3.1. Screening and Optimization of Growth Conditions of Australian Ganoderma Isolates

#### 3.1.1. Stillage Concentration

**Figure 1:**
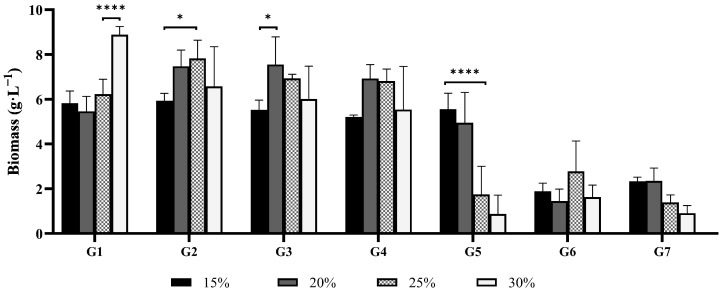
Biomass yields (dry weight) of seven *Ganoderma* isolates cultured in 15%, 20%, 25%, and 30% stillage at the original pH (4.8). Bars represent mean biomass concentration (g·L^−1^) after 10 days of cultivation; Error bars indicate standard deviation among biological replicates (*n* = 3). Statistical differences between treatments for each isolate were determined using one-way ANOVA followed by Bonferroni correction for multiple comparisons (* *p* < 0.05, **** *p* < 0.001).

Wastewaters contain complex organic and inorganic compounds that can inhibit microbial growth [6]. Therefore, an initial screen of seven Australian *Ganoderma* strains was conducted to determine the highest concentration of stillage that would not inhibit fungal growth, as well as the impact of initial pH. The growth of seven *Ganoderma* isolates was assessed at 15%, 20%, 25%, and 30% (*v*/*v*) stillage at the original pH value of 4.8 (Figure 1). Concentrations beyond 30% stillage were excluded, as a pre-screen with other basidiomycete and mold fungi had indicated concentrations above this were inhibitory (Supplementary Materials, Figure S1).

Biomass production of *Ganoderma* isolates varied in response to stillage concentration. Isolates G1 to G4 produced more biomass as the stillage content increased from 15% to 25%. Strains G2, G3, and G4 yielded the highest biomass production concentrations in the range of 20–25% stillage. Stillage at 30% strength significantly improved the biomass yield of isolate G1 (*p* < 0.0001). Biomass production of G2 and G3 significantly increased when the stillage content shifted from 15% to 20–25% (*p* < 0.05), indicating that this concentration was optimal for *Ganoderma* biomass production, while isolates G6 and G7 either grew poorly or were inhibited significantly at concentrations of 25% or higher. The data highlights the importance of optimizing media to ensure inhibitory factors, such as excessive phenolics and complex organic compounds, are not present in excess [7,18]. Operating within a range slightly lower than an inhibitory concentration allows for the least dilution of the residue and highest concentration of assimilable carbon and nitrogen. Considering the experimental data, 25% stillage was determined to be the least-diluted concentration that would allow for favorable growth.

#### 3.1.2. Initial pH

**Figure 2:**
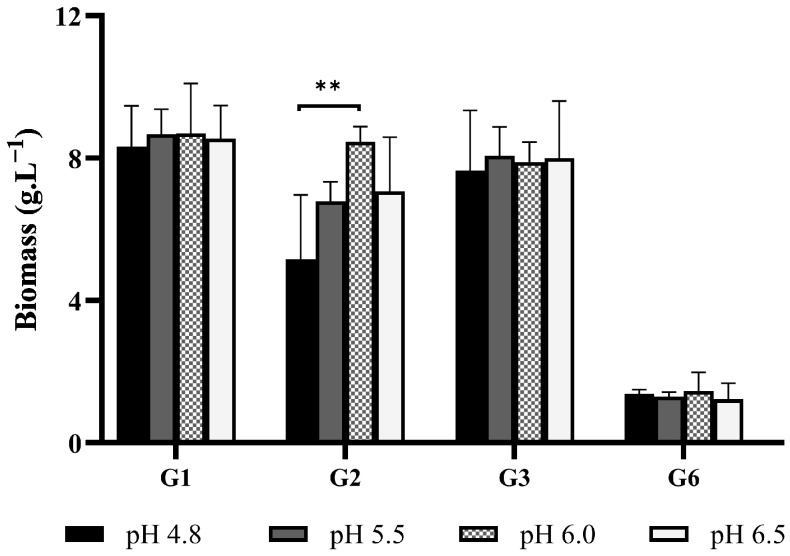
Biomass production of *Ganoderma* isolates (G1, G2, G3, and G6) cultured in 25% stillage medium at an initial pH of either 4.8, 5.5, 6.0, or 6.5. Bars represent mean biomass yield (g·L^−1^) after 10 days of cultivation. Error bars indicate standard deviation from biological replicates (*n* = 3). Statistical differences were assessed using one-way ANOVA followed by Bonferroni correction for multiple comparisons (** *p* < 0.01).

To investigate the impact of initial pH on biomass production, four isolates were assessed in 25% stillage at pH 4.8 (original), 5.5, 6.0, and 6.5. Three isolates were selected based on robust growth in the previous experiment, while a slower-growing isolate was included to determine whether the initial pH had impacted its growth. The initial pH had no significant impact on biomass production for the range assessed, except for G2, which demonstrated a greater biomass yield when the initial pH increased from 4.8 to 6.0 (Figure 2). Growth was relatively consistent for three isolates, with less than 6% variation in biomass production across the pH range. One isolate (G2) averaged slightly lower biomass yields at the more acidic pH values, but these values were minor.

While the acidity of some wastewaters inhibit fungal growth (often due to hyphal dehydration and reduced enzymatic response [7,18]), *Ganoderma* is reported to prefer an acidic-to-near-neutral pH [7,19]. The results align with the studies where a slightly acidic pH benefited *Ganoderma* growth. For example, Hsieh, Hsu, and Yang [11] reported that the optimal biomass production of *Ganoderma lucidum* occurred in thin stillage at a pH of 5.0, while Lebkowska and Zaleska-Radziwll [19] reported an optimum pH range between 4.0 to 5.0. Furthermore, levels below pH 5.0 may enhance biomass production and improve wastewater COD and ammonia removal [3]. As there was generally no significant biomass increase across the pH range assessed, the original pH was used for all subsequent experiments. The three most productive strains were selected for subsequent experiments based on their ability to utilize components of stillage media for biomass production.

#### 3.1.3. Nitrogen Supplement

**Figure 3:**
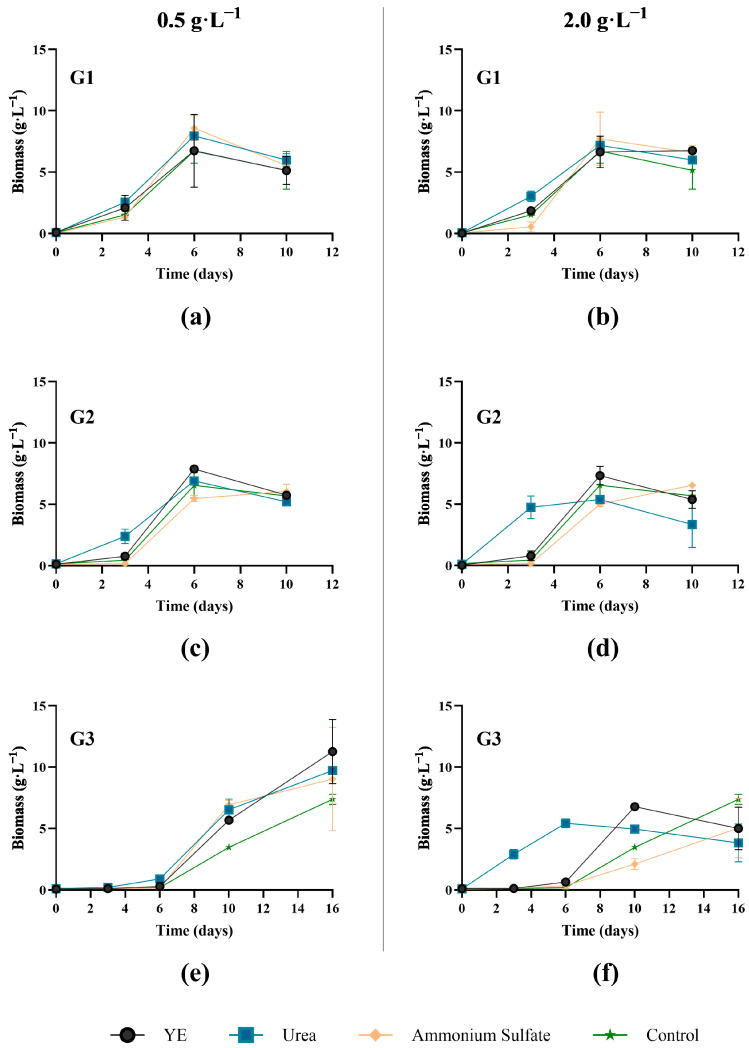
Impact of nitrogen addition on biomass production of three *Ganoderma* isolates (G1 (**a**,**b**); G2 (**c**,**d**); and G3 (**e**,**f**)) in 25% stillage supplemented at 0.5 g·L^−1^ (**a**,**c**,**e**) or 2 g·L^−1^ (**b**,**d**,**f**) yeast extract equivalent. Unsupplemented 25% stillage was used as a control. Error bars indicate standard deviation from biological replicates (*n* = 3).

Stillage often contains sufficient organic carbon to support fungal growth. However, nitrogen supplementation may enhance biomass synthesis when nitrogen is limited or inaccessible. As the complexity of a nitrogenous compound influences growth and protein production in wastewater treatment systems [7], the impact of additional inorganic (ammonium and urea) or complex (yeast extract) nitrogen was determined. Nitrogen supplements were assessed at two levels, with concentrations standardized to the stoichiometric N-equivalent of either 0.5 g·L^−1^ or 2 g·L^−1^ of yeast extract. Data is presented in Figure 3.

In general, similar biomass yields were obtained in the 25% stillage control and N-supplemented stillage, with no significant difference (*p* > 0.05, ANOVA test). This indicated that nitrogen was not a limiting factor. An interesting dose–response behavior was observed for cultures supplemented with urea at 2 g·L^−1^, where an earlier increase in biomass was observed. This was observed between days 3 and 6 for isolates G2 and G3, which displayed a significantly higher biomass yield (*p* < 0.01 for G2, and *p* < 0.0001 for G3) when compared to the lower concentration of urea. Culture supplementation with urea has been reported to enhance biomass production and the nutritional quality of *Ganoderma* [20]. While this enhanced growth could be advantageous for basidiomycetes, such as *Ganoderma* (often relatively slower-growing fungi), it should be noted that the more rapid initial growth was countered by slightly lower total biomass yields for these cultures.

The need to supplement stillage varies, as there is a diverse array of carbon sources used for ethanol production. Some studies have demonstrated significant improvement in biomass production after adding carbon or nitrogen supplements [19], while others have reported high biomass yields without a supplement [21]. In the current study, the data suggests that supplements have negligible impact on biomass yield, other than faster initial growth with a urea supplement. As such, the most pragmatic conditions for more detailed time course analyses of the product were a stillage concentration of 25%, with no pH adjustment or nitrogen supplement.

### 3.2. Ganoderma Biomass Synthesis in Stillage Versus a Nutrient Medium

Three Australian *Ganoderma* isolates were compared in a time course experiment for biomass and product synthesis in 25% rum stillage. This was benchmarked against a nutrient medium containing yeast extract, malt extract, peptone, and glucose. Resultant mycelial biomass was assessed for protein, β-glucan, phenolic, lipid, and antioxidant content over 12 d. The supernatant was assessed for SOC, SN, and total phenolic compound removal.

#### 3.2.1. Mycelial Biomass Synthesis

**Figure 4:**
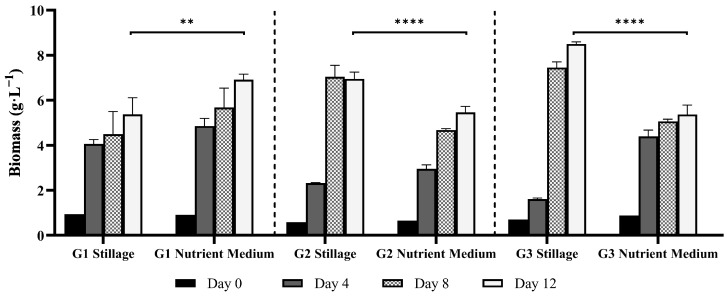
Biomass yields (dry mass) of *Ganoderma* isolates G1, G2, and G3 over 12 days in 25% stillage versus a nutrient medium (yeast extract, malt extract, peptone, and glucose). Bars represent mean biomass concentrations (g·L^−1^) measured on days 0, 4, 8, and 12. Error bars indicate standard deviation among biological replicates (*n* = 3). Statistical comparisons between media types for each isolate were performed using two-way ANOVA with Bonferroni correction for multiple comparisons (** *p* < 0.01; **** *p* < 0.0001).

Isolate G1 grew the most consistently across stillage and the nutrient medium (Figure 4). It also grew the most rapidly, with the greatest amount of biomass produced over the first four days: 4.2 g·L^−1^ in stillage and 4.9 g·L^−1^ in the nutrient medium (*p* < 0.0001 for both). Thereafter, biomass increased gradually, producing 5.4 g·L^−1^ in stillage and 6.9 g·L^−1^ in nutrient medium by day 12 (*p* = 0.0495 in stillage, *p* = 0.0019 in nutrient medium). While G1 produced more biomass in the nutrient medium, G2 and G3 produced more biomass in the stillage medium (*p* < 0.0001 for both). Isolate G2 produced 7.0 g·L^−1^ in stillage and 5.5 g·L^−1^ in nutrient medium, while G3 produced 8.2 g·L^−1^ in stillage and 5.4 g·L^−1^ in nutrient medium. Isolates G2 and G3 produced more biomass in stillage, though they took longer to acclimate than in the nutrient medium, producing the highest rate of biomass between days 4 and 8 in stillage (*p* < 0.0001). In the current study, isolates G2 and G3 grew rapidly in the nutrient medium (notably G3), as would be anticipated in a medium with easily-assimilable carbon and nitrogen sources. The delay observed in stillage medium likely reflects the time required to degrade inhibitory compounds and metabolize complex carbon and nitrogen sources, which is not uncommon in stillage [3]. A similar trend was observed by Ntougias et al. [22], where *Ganoderma* strains degraded phenolic compounds in olive mill wastewater, and biomass increased after an initial lag phase.

Numerous studies have investigated the artificial cultivation of *Ganoderma* using conventional media. Optimal conditions for *Ganoderma* mycelium production generally include glucose as the primary carbon source, an initial pH of 5.0, and incubation temperature of 30 °C [23,24]. Reported yields of *G. lucidum* vary across different conditions: shake flasks (1–10 g·L^−1^), fed-batch fermentation (10–15 g·L^−1^), 10 L stirred-tank bioreactor (10–15 g·L^−1^), and 50 L stirred-tank bioreactor (15–25 g·L^−1^), with some multi-step methodologies yielding up to 30 g·L^−1^ [23,25,26]. Variations within the same scale likely result from differences in media composition and component concentrations. The biomass yields obtained in this study fall within the reported ranges for shake-flask cultures. This outcome demonstrates the potential of alternative *Ganoderma* isolates to perform well in stillage medium.

By-products such as diluted brewer’s spent grain, second cheesy whey, wine distillery effluent, and olive mill wastewater have been used as substrates for *Ganoderma* cultivation [9]. Maximum biomass production reported include 2.2 g·L^−1^ and 3.6 g·L^−1^ for *G. lighzhi* and *G. resinaceum*, respectively, in diluted brewer’s spent grain, and 2.6 g·L^−1^ for *G. frondosa* in wine distillery wastewater. Lower yields, such as 0.5 g·L^−1^ of for *G. lingzhi* and *G. frondosa* in olive mill wastewater, and 0.3 g·L^−1^ for *G. resinaceum* in wine distillery wastewater have also been reported [9]. Notably, biomass production of isolates G2 and G3 in stillage exceeded that observed in the nutrient medium, despite the presence of more complex carbon and nitrogen sources.

#### 3.2.2. Extractable and Crude Protein Content in *Ganoderma* Biomass

**Figure 5:**
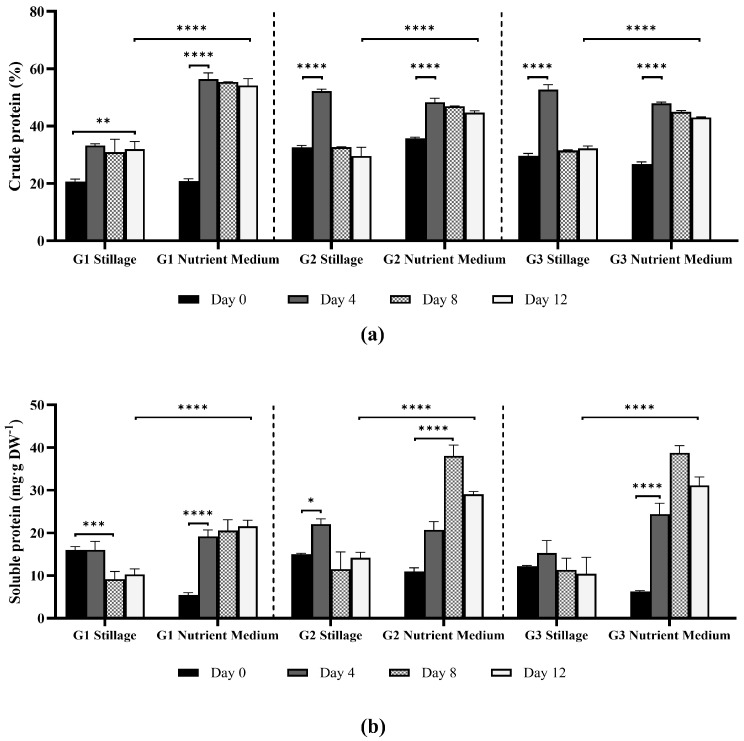
Crude protein (**a**) and internal soluble protein (**b**) content as a function of mycelial biomass of *Ganoderma* isolates (G1, G2, and G3) grown in 25% stillage or nutrient medium (yeast extract, malt extract, peptone, and glucose). Crude protein (% of dry weight) was estimated by multiplying nitrogen percentage by a conversion factor of 6.25. Soluble protein (mg·g^−1^ dry weight) was quantified following Bradford assay using a BSA standard. Error bars indicate standard deviation among biological replicates (*n* = 3). Statistical comparisons between media types for each isolate and time point were performed using two-way ANOVA followed by Bonferroni correction for multiple comparisons (* *p* < 0.05, ** *p* < 0.01, *** *p* < 0.001, and **** *p* < 0.0001).

Crude protein content was estimated based on total nitrogen, while extractable biomass protein (i.e., soluble in aqueous solution) was quantified using the Bradford assay (Figure 5). Crude protein is a measure of all nitrogen compounds (including structural components), while extractable proteins represent the metabolic active fractions, such as enzymes, involved in primary metabolism, stress response, and signaling pathways [27].

Protein synthesis was greater in the nutrient medium (*p* < 0.0001, ANOVA test). This would be expected due to its content of high-quality nitrogenous compounds in malt extract, yeast extract, and peptone. Extractable protein from biomass in the nutrient medium increased steadily for isolates G2 and G3 until day 8 (38.0 and 38.7 mg·g^−1^, respectively), and declined at day 12 as the fungi entered the stationary phase. In stillage, extractable protein content peaked at day 4 for all isolates, yielding 16.0, 22.1, and 15.3 mg·g^−1^ for isolates G1, G2, and G3 respectively. This correlated to nitrogen removal from stillage (Section 3.3.1).

Crude protein increased rapidly by day 4 in all media (*p* < 0.01 for G1 in stillage; *p* < 0.0001 for G2 and G3) and remained high (>40%) until day 12 for the nutrient medium. In stillage, the highest crude protein content was obtained by day 4, with maximum yields of 52.8% ± 1.2 for G3, 52.2% ± 0.5 for G2 and 33.3% ± 0.4 for G1. In the nutrient medium, the isolates yielded 56.4% ± 1.5 (G1), 48.4% ± 1.0 (G2), and 47.9% ± 0.3 (G3) crude proteins. The decrease in crude protein after the fourth day likely reflects metabolic shifts due to nutrient limitations leading to lower protein accumulation, or the rapid accumulation of storage compounds, such as carbohydrates and lipids, reducing the total nitrogen content relative to biomass [28].

Proteins are abundant in *Ganoderma* species and can serve diverse functions, including structural (cell wall proteins), extracellular enzymatic activity (proteases, lipases, laccases, and peroxidases), biosynthesis of secondary metabolites, defense mechanisms, stress response, signal transduction, and transcriptional or post-transcriptional regulation [29]. The high protein content in fungal mycelia supports their potential use as an alternative protein source [30]. A broad range of protein content is reported in the literature for fruiting bodies of edible and medicinal fungi, and rarely on mycelium content. Nitrogen source strongly influences these values and is a key component in the production of proteins, lipids, and carbohydrates [30]. Mycelial biomass of the edible mushrooms *Pleurotus ostreatus* and *Lentinula edodes* contained 26% and 53% crude protein, respectively, when cultured in generic growth media [31,32]. In this study, isolate G1 produced the highest crude protein (56.4% ± 2.1) in the nutrient medium after four days. Overall, studies report high (>30%) crude protein content in edible and medicinal mushrooms, such as *P. ostreatus, L. edodes, A. biosporus*, and *T. versicolor* [31], or surpass 50% crude proteins for the same species [30].

The global leader in commercial fungal products from mycelium is Quorn™. Their mycoprotein products from *Fusarium venenatum* have been available since 1985 and have regulatory approval in the EU, the USA, Australia, and other jurisdictions [33]. The product is rich in in dietary fiber (notably chitin and β-glucans) and report a 4–10-fold lower carbon footprint than meat [33]. Market studies forecast global mycoprotein revenue to increase from USD 805 million in 2025 to approximately USD 1.46 billion by 2034. It is Generally Recognized as Safe (GRAS) and there is an increasing consumer demand for sustainable protein [34].

#### 3.2.3. Antioxidant Activity and Phenolic Content of Ganoderma Extracts

**Figure 6:**
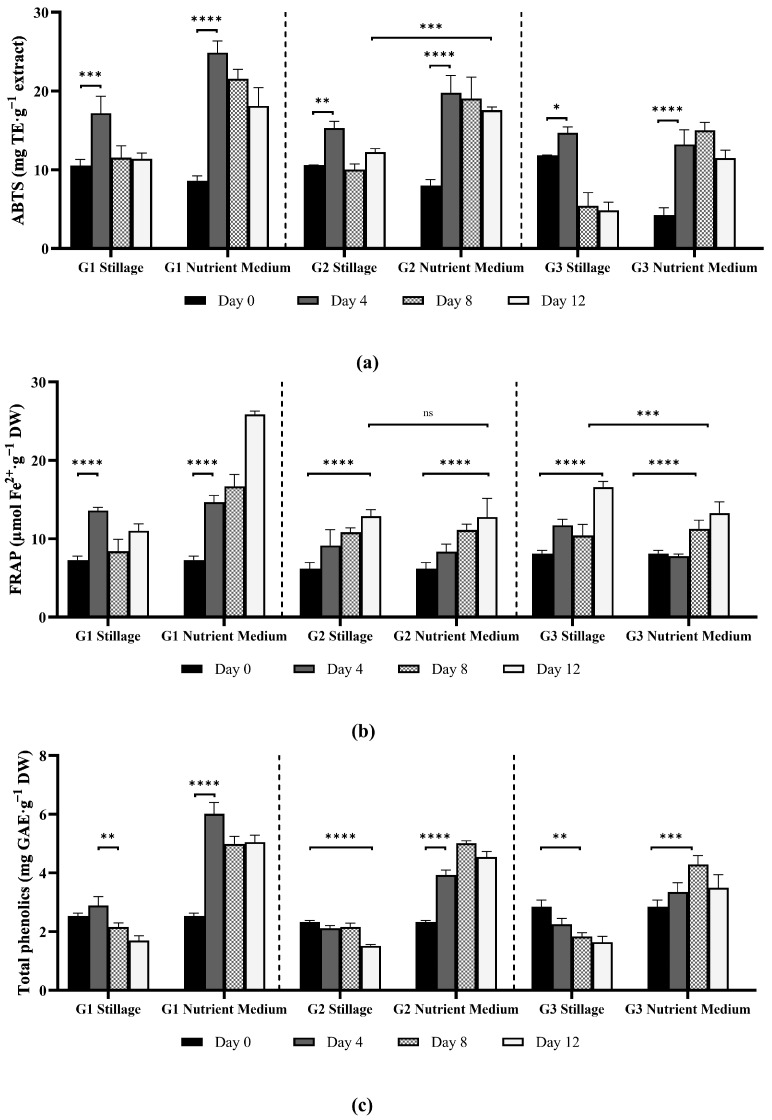
ABTS antioxidant activity (**a**), FRAP (**b**), and total phenolic compounds (**c**) of *Ganoderma* isolates G1, G2, and G3, grown in 25% stillage and nutrient medium (yeast extract, malt extract, peptone, and glucose). Bars represent mean values at days 0, 4, 8, and 12. Error bars indicate standard deviation from biological replicates (*n* = 3). Statistical comparisons between media type and time points for each isolate were performed using two-way ANOVA with Bonferroni correction for multiple comparisons (* *p* < 0.05, ** *p* < 0.01, *** *p* < 0.001, and **** *p* < 0.0001; ns = not significant). Significant differences between the two media were observed on the final day for all assays and isolates (*p* < 0.0001), except for G2 in ABTS, and G2 and G3 in FRAP, as indicated.

The data for the ABTS and FRAP assays, as well as the total phenolic compounds, is presented in Figure 6. The ABTS assay is a widely recognized method for evaluating the hydrogen-donating capacity of hydrophilic and lipophilic antioxidants [35], while the ferric reducing antioxidant power (FRAP) assay is a measure of the capacity to reduce ferric (Fe^3+^) to ferrous (Fe^2+^) ions [36]. These assays are commonly used together as they assess distinct but complementary antioxidant mechanisms, aligning with the functional role of natural antioxidants in biological systems. Antioxidant activity frequently correlates positively with phenolic compound content.

##### ABTS Assay

Isolates cultivated in nutrient medium recorded higher ABTS antioxidant activity than those in 25% stillage (Figure 6a). Maximum ABTS values in nutrient medium reached 24.9, 19.8, and 15.0 mg TE·g^−1^ for isolates G1, G2, and G3, respectively, while values in stillage peaked at 17.2 (G1), 15.3 (G2), and 14.7 mg TE·g^−1^ (G3). Antioxidant activity increased rapidly by day 4 and declined over time. This decline was more pronounced in stillage cultures than in nutrient medium and likely reflects response to nutrient depletion and shifts in fungal metabolism with the subsequent degradation of antioxidant compounds [37,38]. The antioxidant activity varied among isolates. Isolate G1 recorded the highest antioxidant activity and phenolic content among isolates. Strain-specific differences in antioxidant activity may be linked to the isolate’s response to media conditions and its production of polysaccharides, proteins, organic acids, pigments (carotenoids), alkaloids, flavonoids, and other phenolic compounds, known for their antioxidant capacity [36,38].

*Ganoderma* have shown antioxidant activity in purified extracts, ranging from 9.46 mg TE·g^−1^ and 8.59 mg TE·g^−1^ in *G. resinaceum* and *G. pfeifferi*, respectively, to as high as 170.32 mg TE·g^−1^ in *G. pfeifferi* extracts [39,40]. Cadar et al. [41] found 20.3 mg TE·g^−1^ in extract from *G. lucidum*. This study analyzed the antioxidant activity and phenolic content of freshly prepared mycelial hot-water extracts, differing from other studies that measured this activity in concentrated extracts. While direct measurement avoids additional processing steps that could alter the antioxidant activity, such as drying and resuspending extracts, it may yield lower values as activity is expressed per gram of dry mycelium, as opposed to activity per gram of extract [42]. Nonetheless, values obtained for isolates G1, G2, and G3 were comparable to reported values for *Ganoderma*.

##### FRAP Assay

Isolates grown in nutrient medium generally exhibited higher FRAP activity than in stillage, except for isolate G2, which displayed no significant difference between media (Figure 6b). Specifically, the maximum FRAP values in nutrient medium reached 25.9, 12.8, and 13.3 µmol Fe^2+^·g^−1^ for isolates G1, G2, and G3, respectively. In stillage, the highest yields were 13.6, 12.9, and 16.6 µmol Fe^2+^·g^−1^ for the same isolates. This trend aligns with the ABTS data, indicating that nutrient medium favors antioxidant compound synthesis. The cultivation environment significantly influences metabolic pathways and secondary metabolite production in fungi, thereby affecting its antioxidant activity [43].

Fungal extracts with high FRAP values may provide health benefits to consumers by reducing oxidative stress and mitigating associated chronic diseases [40]. Reported FRAP values vary across species, substrates, and extraction methods, with highly variable data according to those conditions and the fungus’ metabolism. For example, Tan et al. [44] observed distinct FRAP activity in *G. lucidum* when comparing mycelium from solid-state fermentation (0.07–0.11 μmol Fe^2+^·μg^−1^ extract) to submerged fermentation (0.06–0.21 μmol Fe^2+^.μg^−1^ extract) and fruiting body (0.36–0.76 μmol Fe^2+^·μg^−1^ extract). Other studies reported 0.523 mmol Fe^2+^·g^−1^ for *G. lucidum* antler-type fruiting bodies, and 0.09–0.13 mmol Fe^2+^·g^−1^ in the fruiting body after different drying methods [45,46]. Extracts from *G. applanatum* produced 107.5 μmol Fe^2+^·g^−1^, while *G. lucidum* recorded a FRAP of 38.8 μmol Fe^2+^·g^−1^ [47]. In the current study, the three isolates produced antioxidant compounds under stillage and nutrient conditions, with very similar values among the two media for isolates G2 and G3, even though carbon and nitrogen sources in stillage are often complex and not readily available [43,48].

There is substantial interest in incorporating natural antioxidants in food preservation, nutraceuticals, pharmaceuticals, and cosmetics [38,49]. *Ganoderma* antioxidants show anti-inflammatory and immune system enhancement effects and potentially mitigate oxidative stress-related conditions, such as cardiovascular and neurodegenerative diseases [36,38,40]. The use of industrial by-products, such as stillage, offers a viable substrate for mycelial production. The antioxidant activity observed in this study is consistent with the values previously reported for *Ganoderma* species, with cultivation in nutrient medium supporting a higher radical-scavenging capacity among isolates G1, G2, and G3 compared to stillage cultures, thereby underscoring the influence of substrate type on antioxidant potential.

##### Phenolic Compounds in *Ganoderma* Biomass

Isolates cultivated in nutrient medium showed a significantly higher (*p* < 0.0001, ANOVA test) total phenolic content than those grown in stillage (Figure 6c). In stillage, the phenolic content of isolate G1 peaked at 2.9 mg GAE·g^−1^, whereas G2 and G3 reached 2.2 and 2.3 mg GAE·g^−1^, respectively. In nutrient medium G1 recorded 6.0 mg GAE·g^−1^ on day 4 (*p* < 0.0001), while G2 and G3 peaked at 5.0 and 4.3 mg GAE·g^−1^, respectively, at day 8. Subsequently, G1′s TPC declined on day 8, aligning with G2 by day 12. The variable total phenolic content among different media and isolates highlights the influence of substrate composition and strain variations in the production of phenolics.

Phenolic compounds are important defense and signaling compounds [50]. They possess antioxidant, immunomodulatory, and anti-inflammatory effects and occur in both mycelia and fruiting bodies [38]. Phenolics yield is significantly impacted by the extraction method and varies considerably in the literature [49]. Raseta et al. [40] reported TPC ranging from 50.9 to 265.4 mg GAE·g^−1^ in *G. applanatum*, depending on the extraction solvent used, while supercritical CO_2_ extraction yielded a range of 41.3–63.8 mg GAE·g^−1^ from *G. lucidum* [36]. Extracts of *G. lucidum* obtained under different drying conditions yielded 2.5 to 4.7 mg GAE·g^−1^ [45]. In this study, phenolic compounds were quantified from aqueous extracts, which can contain polar phenolics, like phenolic acids, but are generally less efficient in recovering non-polar phenolics [51].

Phenolic compounds contribute to fungal defense and antioxidant capacity by reducing oxidative stress [38,52]. *Ganoderma* strains contain a broad array of phenolics—including phenolic acids (p-hydroxybenzoic, protocatechuic, gallic, caffeic, and vanillic) and flavonoids (quercetin, myricetin, hesperetin, and apigenin) [52]. The wide diversity in *Ganoderma* phenolics support its potential as a source of bioactive metabolites for functional foods, nutraceuticals, and topical formulations [38]. Numerous studies, including the present one, have observed a strong positive association between phenolic content and antioxidant activity. Other molecules, such as polysaccharides and proteins, also contribute to the antioxidative properties of *Ganoderma* [37,52].

When cultivated in 25% stillage, *Ganoderma* isolates demonstrated distinct antioxidant and phenolics profiles. Isolates G2 and G3 exhibited strong positive correlations between FRAP activity and β-glucan content (G2, r = 1.0; G3, r = 1.0, *p* = 0.005). In G3, the phenolic content negatively correlated to soluble organic carbon removal (r = −1.0), phenolics in stillage (r = −1.0), and total nitrogen in stillage (r = −0.9). Additionally, FRAP increased with biomass in all isolates (r = 0.6–0.9), while the phenolic content decreased as biomass increased (r = −0.5 to −0.9). However, these trends lack statistical significance.

Isolate G1 exhibited the highest ABTS activity on day 4 (17.9 mg TE·g^−1^), but this did not differ significantly from isolates G2 and G3. In contrast, G3 recorded the highest FRAP activity on day 12 (16.6 µmol Fe^2+^·g^−1^), significantly exceeding values of G1 (*p* < 0.0001) and G2 (*p* < 0.01). Isolate G1 also had the highest total phenolic content on day 4 (2.9 mg GAE·g^−1^), surpassing G2 (*p* < 0.001) and G3 (*p* < 0.05). Overall, isolate G1 was the most consistent across the ABTS and phenolic assays, while G3 displayed superior FRAP activity.

#### 3.2.4. Lipids in *Ganoderma* Biomass

**Figure 7:**
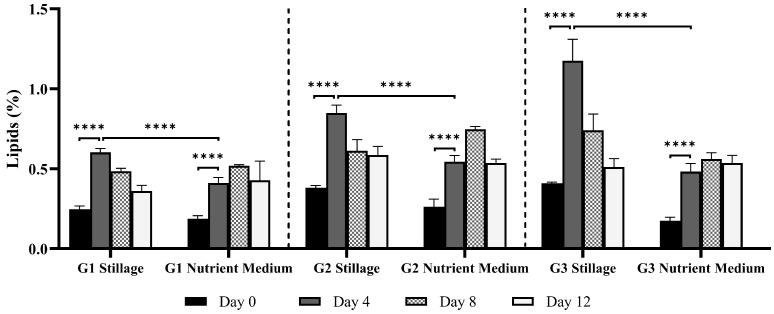
Mycelial lipid content (% of dry weight) of *Ganoderma* isolates G1, G2, and G3, cultured in 25% stillage and nutrient medium (yeast extract, malt extract, peptone, and glucose) over 12 days. Bars represent mean lipid content measured on days 0, 4, 8, and 12. Error bars indicate standard deviation from biological replicates (*n* = 3). Statistical comparisons between time points for each medium and isolate were performed using two-way ANOVA with Bonferroni correction for multiple comparisons (**** *p* < 0.0001).

Stillage medium supported significantly higher lipid production than nutrient medium (Figure 7), with lipid content peaking early (*p* < 0.0001, Day 4) in stillage and reducing afterward. Maximum recorded values in stillage were 0.6, 0.8, and 1.2% for isolates G1, G2, and G3, respectively. The decrease in lipids may be linked to a shift in the fungus metabolism from lipid storage to biomass production, a phenomenon reported for *G. lucidum* and supported by the pronounced exponential growth between day 4 and day 8 [53]. In nutrient medium, lipid content was higher at day 8 for all isolates, reaching 0.5, 0.7, and 0.6% for G1, G2, and G3, respectively. Correlation analysis of nutrient medium data revealed that lipid accumulation associated strongly with β-glucan content in G1 (r = 1.0) and positively correlated with both β-glucan (r = 0.6) and soluble proteins (r = 0.9) in G2. For G3, lipid content had a strong positive correlation with ABTS activity (r = 0.8) and soluble proteins (r = 0.9). Lipid content in fungi can range from 0.6 to 85% of dry weight, depending on factors such as species, sample type, and cultivation conditions [54]. *Ganoderma lucidum* can yield up to 2.35% lipids in mycelia [53], 1.52–5.8% in fruiting bodies [41], and as high as 40% lipids in spores [55].

In many fungi, lipid levels decline over extended cultivation (even when carbon is not limited), reflecting a shift from storage to biomass growth or sporulation [54]. Parameters such as aeration, agitation, incubation duration, and extraction protocols are known to affect both overall yield and lipid composition [26,53]. Palmitic, stearic, oleic, linoleic, and linolenic acids are frequently observed in fungal extracts, with unsaturated species often dominating [56]. In *G. lucidum*, oleic, palmitic, and linoleic acids can constitute up to 88% of the total fatty acids, while the major sterols, such as ergosterol and ergosta-7,22-dien-3β-ol, may represent approximately 80% of total sterols [55]. Previous work on *Ganoderma* has predominantly measured lipid content in fruiting bodies or spores, whereas mycelial lipids are relatively unreported [53]. To the best of our knowledge, the lipid content of Australian *Ganoderma* strains has not been previously documented.

#### 3.2.5. β-Glucan Content in *Ganoderma* Mycelia

**Figure 8:**
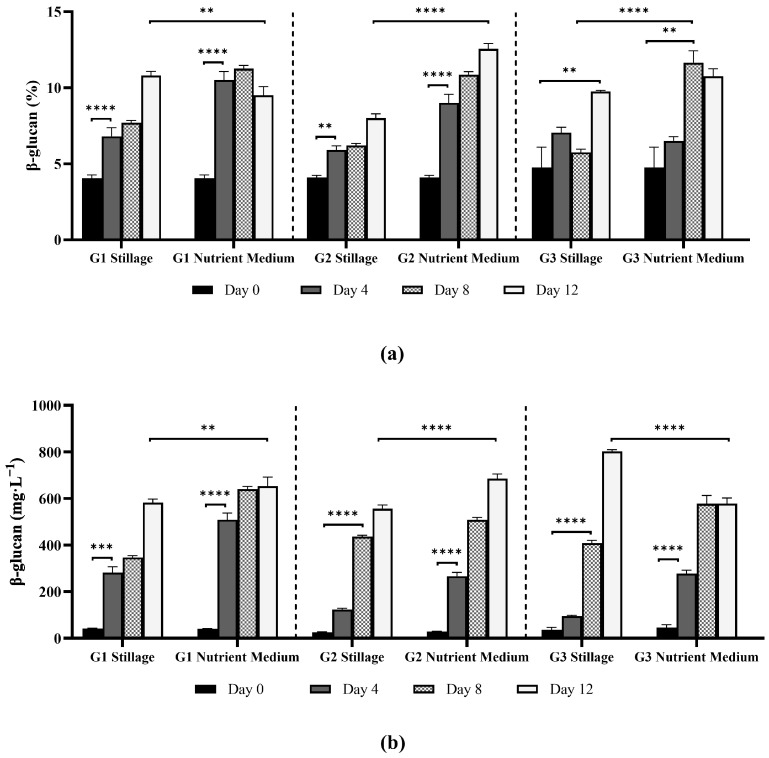
β-glucan content in biomass (**a**) and volumetric β-glucan (**b**) yield data of isolates G1, G2, and G3 grown in either 25% stillage or nutrient media (yeast extract, malt extract, peptone, and glucose). Bars represent mean values of β-glucan content measured on days 0, 4, 8, and 12. Error bars indicate standard deviation from biological replicates (*n* = 3). Statistical comparisons between time points and media for each isolate were performed using two-way ANOVA followed by Bonferroni correction for multiple comparisons (** *p* < 0.01, *** *p* < 0.001, and **** *p* < 0.0001).

In this study, β-glucan content exceeded 10% of biomass from both media (Figure 8). Generally, β-glucan content increased with incubation time for all isolates (*p* < 0.0001, ANOVA test), with a slight reduction after day 8 in nutrient medium (*p* < 0.05 for G1; non-significative for G3). Mycelial β-glucan content increased faster in the nutrient medium. By day 12, G1 and G3 stillage cultures reached >100 mg·g^−1^ β-glucan, while G2 yielded up to 125 mg·g^−1^ β-glucan. Timm et al. [7] observed a similar trend for submerged *G. lipsiense* cultures using brewery wastewater: β-glucan content slightly decreased after an early increase, maintaining most of its content until the end of experimentation.

Volumetric production of β-glucans is an important consideration as the yield per liter strongly impacts the viability of a production system. Although nutrient medium supported higher β-glucan content, stillage yielded more biomass, compensating for the lower metabolite production. Isolate G3 achieved the highest β-glucan volumetric yield in stillage (802 mg·L^−1^) and surpassed nutrient medium (685 mg·L^−1^). In contrast, nutrient medium consistently supported higher volumetric yields for G1 and G2, yielding 653 and 685 mg·L^−1^ as opposed to 582 and 555 mg·L^−1^ in stillage, respectively. The increased biomass production in stillage helps compensate for the lower production of β-glucans, with isolate G3 demonstrating greater β-glucan production per liter of substrate in stillage. Ultimately, the use of waste streams like stillage to produce valuable compounds, such as *Ganoderma* β-glucans, supports the biorefinery concept and generates valuable bioactive compounds while reducing environmental impact of a residue.

β-glucans are key structural components in *Ganoderma* cell wall and are responsible for immunomodulatory, anti-inflammatory, and antitumor effects in humans [57]. While plant-derived β-glucans are typically linear with β-(1→3) and β-(1→4) bonds, fungal β-glucans possess a β-(1→3)-linked backbone with β-(1→6) side chains that form a triple-helix structure critical to their bioactivity [58]. These structures form viscous solutions in the gastrointestinal tract, which can delay gastric emptying and glucose absorption, leading to reduced postprandial blood glucose levels and improved lipid profiles. Consequently, β-glucans are associated with cardiovascular and metabolic health benefits [59]. In contrast, the branched structure of fungal β-glucans is recognized by specific immune receptors, including Dectin-1 and complement receptor 3 (CR3), leading to immunomodulatory effects. Fungal β-glucans can activate macrophages, natural killer cells, and other components of the innate immune system, contributing to their potential anticancer and anti-infective properties [60]. *Ganoderma* polysaccharide products often guarantee a minimum β-glucan concentration, which scales with price. Products rich in β-glucans exceed USD 12,000/kg, while cosmetics- or food-grade bulk extracts can sell for USD 1,200/kg depending on purity—typically in the range of 5–10% [61].

A wide range of β-glucan contents have been reported in *Ganoderma* mycelia, reflecting differences in species, strains, and cultivation conditions. Suwanno and Phutphat [62] obtained 58 mg·g^−1^ β-glucans in *Ganoderma* sp. grown on potato dextrose agar, while Papaspyridi et al. [25] reported 9.5% β-glucans of *G. australe* cultured in a bioreactor. Additionally, 9.0% of β-glucans was reported in the mycelia of *G. calidophilum*, while *G. dahlia* yielded 7.1% and *G. valesiacum* 5.6% β-glucans [62]. Timm et al. [7] reported 23.9% in *G. lipsiense* cultivated supplemented brewery wastewater. Genetic variation among strains [63], culture conditions, including pH, temperature, and carbon and nitrogen sources, impact yield [64].

Fungal β-glucans can be used in cosmetic formulations, functional foods, and nutraceuticals due to their physicochemical and beneficial health properties [65]. Furthermore, their solubility and viscosity support their use as thickening agents, stabilizers, and emulsifiers [65]. Pharmaceutical applications are largely based on their immuno-modulatory activity, including stimulation of innate immunity and inflammation regulation [65]. Commercial specifications typically require β-glucan purity ≥ 80%, with extraction for animal feed often allowing lower purity requirements [65]. Formulation concentrations vary by application: meat emulsions (1.5–4%), yogurt (0.4–2.5%), breads (5.5–20%), noodles (10%), soups (2%), and dairy (≤2.5%) [65]. Notable commercial products include cosmetics such as iUNIK, a moisturizer cream, and nutraceuticals, like Immulink MBG, a 70% mushroom β-glucan concentrate [65,66]. Downstream processing is hampered by the high viscosity of β-glucan-rich extracts, which hinders filtration, pumping, and drying of extracts. Purification costs further escalate since color removal, enzymatic digestion, dialysis, and ultrafiltration are often required to reach the purity threshold [65]. In general, stillage supported *Ganoderma* β-glucan production, with yields of 100 mg·g^−1^ (10% *w*/*w*). Similar β-glucan contents were synthesized in both media, but production was faster in the nutrient medium. However, promoted higher biomass production stillage ultimately resulted in a greater volumetric yield. Among the isolates, G3 outperformed G1 and G2 with respect to volumetric β-glucan production. Yields up to 13% and 802 mg·L^−1^ were recorded, matching or surpassing values for *Ganoderma* grown in submerged cultures [25,62]. These findings demonstrate the capacity of *Ganoderma* strains to effectively utilize stillage as a nutrient source for β-glucan synthesis, potentially offering a cost-effective and sustainable alternative to conventional media. The use of agro-industrial residues, such as stillage, not only supports similar or superior yields to those reported in submerged cultivation systems but also aligns with biorefinery principles by converting low-value waste streams into high-value functional metabolites.

### 3.3. Bioremediation Potential of Ganoderma in Stillage

#### 3.3.1. Removal of Phenolic Compounds

**Figure 9:**
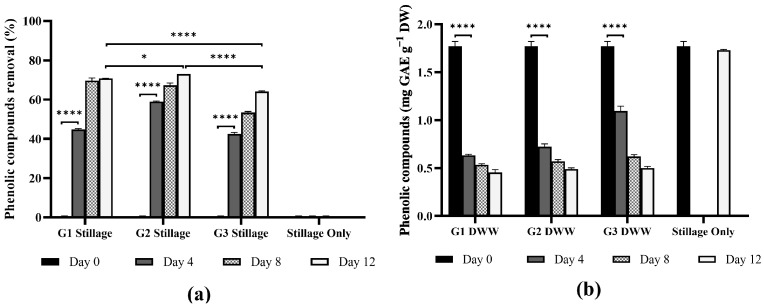
Removal of phenolic compounds by *Ganoderma* isolates G1, G2, and G3 over 12 days in 25% stillage as (**a**) phenolic removal capacity (% of initial concentration) and (**b**) total phenolic compounds (mg·g^−1^ dry weight, expressed as gallic acid equivalents) in 25% stillage during *Ganoderma* culture. Stillage Only represents an abiotic control. Bars represent mean values taken on days 0, 4, 8, and 12. Error bars indicate standard deviation from biological replicates (*n* = 3). Statistical comparisons were performed using two-way ANOVA with Bonferroni correction for multiple comparisons (* *p* < 0.05 and **** *p* < 0.0001).

*Ganoderma* isolates demonstrated effective degradation of phenolic compounds in stillage (Figure 9). Isolates G1 and G2 removed >60% phenolics in four days (*p* < 0.0001). All isolates degraded 72–74% of the phenolic compounds over 12 days, differing significantly from the abiotic control (*p* < 0.0001), which decreased by 3% (attributed to natural oxidation). This is notable considering the high starting phenolics concentration of 1.8 g·L^−1^.

Phenolic compounds in stillage originate from the lignin and other polyphenolic substances present in the substrates used for alcohol production [67]. The composition and concentration of these polyphenols, including phenolic acids and flavonoids, vary depending on the raw material, processing methods, and storage conditions [67]. Stillage often contains a complex mixture of phenolic acids (e.g., hydroxy-benzoic acid, vanillic acid, ferulic acid, synaptic acid, and p-coumaric acid) and flavonoids [67]. White-rot fungi, such as *Ganoderma* oxidize phenolic compounds, via lignolytic enzymes, such as laccase, which form quinones or unstable radicals that polymerize and precipitate from solution, facilitating their removal by filtration or flocculation [68].

The efficacy of phenolic compound removal by *Ganoderma* varies significantly, influenced by fungal species and wastewater type. Koutrotsios and Zervakis [69] reported a 61% phenolic removal for *G. carnosum* in 25% olive mill wastewater, while Matos et al. [70] reported 94% removal by *G. applanatum* cultures in 20% olive mill wastewater. Ntougias et al. [22] reported 75% phenolics removal in *G. australe* cultured in 25% olive mill wastewater, while *Ganoderma* sp. removed 95% phenolics in the same wastewater [48]. Although *G. resinaceum* was inhibited by more than 50% in olive mill wastewater containing 1.5 g·L^−1^ phenolics [10], the strains in the current study performed well—even with an initial phenolics load of 1.8 g·L^−1^.

#### 3.3.2. Removal of Soluble Organic Carbon (SOC) and Soluble Nitrogen (SN)

**Figure 10:**
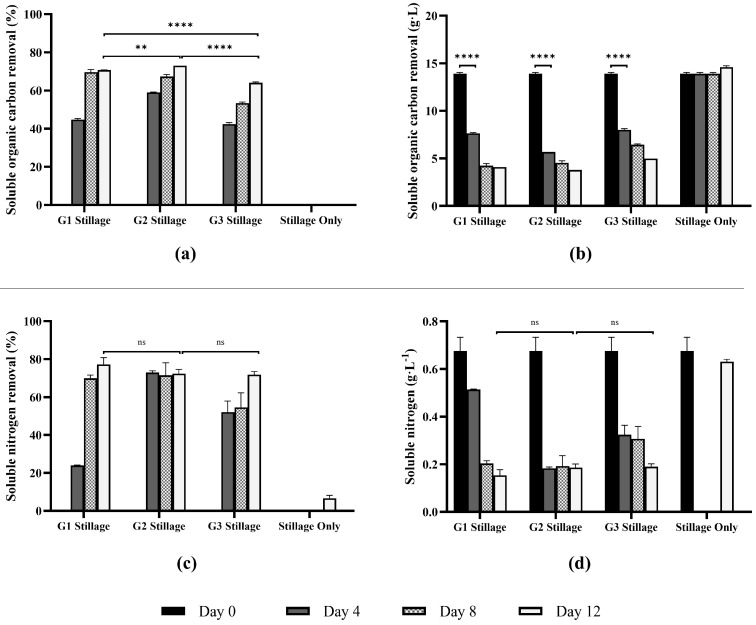
Removal of soluble organic carbon (**a**,**b**) and soluble nitrogen (**c**,**d**) from stillage by *Ganoderma* isolates G1, G2, and G3 grown for 12 d. Stillage only represents an abiotic control. Bars represent mean values measured on days 0, 4, 8, and 12. Error bars indicate standard deviation among biological replicates (*n* = 2). Statistical comparisons were conducted using two-way ANOVA with Bonferroni correction for multiple comparisons (** *p* < 0.01; **** *p* < 0.0001); ns = not significant.

In this study, *Ganoderma* isolates G1, G2, and G3 were evaluated for their ability to reduce SOC and SN in stillage (Figure 10). The initial SOC of ~14,000 mg·L^−1^ was rapidly reduced during the first four days of fungal growth (G1: 45%, G2: 59%, and G3: 42%, *p* < 0.001). Between days 4 and 12, all three *Ganoderma* isolates continued to reduce SOC, though at a slower rate, ultimately removing 64 to 73% of the SOC by day 12. The slower rate reflects the degradation of more complex and recalcitrant carbon compounds [6]. By day 12, all isolates achieved significantly higher SOC than the uninoculated controls (*p* < 0.001).

Fungi can metabolize soluble carbon using multiple enzymatic mechanisms, converting it into biomass, cell products or mineralizing it to CO_2_ [71]. *Ganoderma* and other white-rot fungi can degrade organic compounds into different wastewater matrices [72]. For example, *Ganoderma resinaceum*, *Irpex lacteus*, *Trametes versicolor*, and *Phlebia rufa* grown in winemaking by-products reduced >80% organic carbon [6], while *T. versicolor* was capable of 50% removal in agricultural wastewater [73]. In this study, initial SOC levels were ~14,000 mg·L^−1^, which is within the range reported in previous studies (5895 mg·L^−1^ in rum stillage [74] and 22,200 mg·L^−1^ in molasses-based stillage [75]). These values typically fluctuate according to feedstock composition and seasonal variations, highlighting the importance of flexible, robust treatment options.

Initial nitrogen levels of 675 mg·L^−1^ were reduced by up to 73 ± 0.6% after four days of culture (G2). Both G2 and G3 consumed over 50% of the soluble nitrogen by day 4, while G1 removed comparable levels by day 8. By day 12, nitrogen removal ranged from 72 ± 1.2% to 77 ± 2.5%, while the abiotic control decreased only 6.7% ± 1.0, significantly differing from G1, G2, and G3 cultures (*p* < 0.0001). Rapid nitrogen uptake during the early growth likely led to nitrogen depletion, restricting biosynthetic processes, such as protein synthesis, which require sustained nitrogen availability [76].

Stillage treatment is traditionally centered around anaerobic digestion and bacterial consortia [1]. Anaerobic systems remove 60–85% soluble organic carbon, and may exceed 95% efficiency with membrane retention, while also generating biogas for on-site energy [1]. However, a dark effluent rich in ammoniacal nitrogen and elevated COD remains, making post-treatment essential [1]. Bacterial consortia can further reduce COD by 50 to 70% within 2 to 5 days and are easily scalable, though they rarely degrade recalcitrant melanoidins completely [1].

Subsequent detoxification may involve algae and fungi. White-rot fungi, such as *Trametes versicolor*, *Pleurotus sajor-caju*, and *Aspergillus oryzae*, can remove 50 to 86% COD over 7 to 25 days [6,72]. These organisms can outperform bacterial systems but require longer treatment times [6]. Algal–bacterial consortia applied to anaerobic effluents can remove 50–90% residual COD and 70–100% ammoniacal nitrogen, but they require dilution, continuous sunlight, and large land area [1]. Overall, while the current bioremediation strategies using anaerobic digestion and bacteria consortium effectively remove bulk organics, additional fungal or algal treatments may be necessary to remove persistent pollutants [1]. No single method delivers a comprehensive and balanced removal or organic matter, phenolics, melanoidins, and other stillage-derived compounds.

Recent studies have demonstrated that *Ganoderma* spp. can achieve high removal efficiencies in controlled systems: *G. lucidum* mycelial pellets removed ~96% of COD and 93% of ammoniacal nitrogen from synthetic sewage [77], while a Malaysian *G. lucidum* strain produced 95–100% ammonia and COD within 30 h [78]. Similarly, *G. resinaceum* reduced total phenolic content and TOC by over 80% in wine distillery vinasse, a performance comparable to that of white-rot fungi commonly employed in bioremediation [6]. However, scaling fungal systems remains challenging. Fungal bioreactors (including suspended pellets or immobilized biofilms) require aeration and controlled pH and temperature (typically 25–30 °C) to sustain fungal activity. Compared to bacterial systems, fungal biomass yields are lower and growth rates slower. Notably, synergistic effects are observed in combined fungal–bacterial systems [79]. Nonetheless, when optimized, fungal systems have achieved high removal efficiencies within relatively short time (1–4 days) [78].

Fungi can rapidly consume nitrogen in waste streams, especially if readily available ammonia or glutamate are present in the media [80]. Stillage streams may contain soluble nitrogen levels in a range from 262 mg·L^−1^ in rum stillage [74], and 600–1470 mg·L^−1^ in sugarcane stillage [81], though it may vary according to substrate. This nitrogen is present as a combination of organic and inorganic compounds, including ammonium, and nitrite [82,83]. The reduction SN shoed after *Ganoderma* cultivation is primarily attributed to the fungal absorption and assimilation of nitrogenous compounds, which are incorporated into cellular components, such as proteins and nucleic acids, to support biomass growth [80], or excretion of extracellular enzymes [83]. The abiotic control decreased by 6.7% ± 1.0 over 12 days.

Although the focus of this work was primarily on *Ganoderma* metabolite production, the simultaneous reduction in organic and nitrogen loads presents a valuable co-benefit. Biomass production positively correlated with removal of organic carbon, nitrogen, and phenolics compounds from stillage (r > 0.8 for all isolates). Isolate G2 had a slightly lower correlation between biomass and nitrogen removal (r = 0.7). Overall, the results demonstrate the effectiveness of *Ganoderma* isolates G1, G2, and G3 in substantially reducing both SOC and TN in stillage. On average, the isolates were able to remove 70% of the soluble organic carbon, and 74% of soluble nitrogen. Isolate G2 appeared better-suited for faster organic carbon and nitrogen removal from stillage. In view of the expanding interest in sustainable wastewater treatment solutions, *Ganoderma*-based systems could offer a promising alternative that integrates resource recovery with environmental remediation, thereby contributing to more ecologically responsible industrial processes.

The *Ganoderma* research has traditionally focused on its bioactive metabolites—particularly polysaccharides and triterpenoids—for medicinal and nutraceutical applications. The use of *Ganoderma* species for bioremediation is relatively recent and differs in function and design from its traditional. In aqueous effluents, *Ganoderma* species produce lignin-modifying enzymes and adsorption capacity that facilitates degradation of a broad range of recalcitrant organic pollutants [84]. While prior studies have applied *Ganoderma* to various wastewaters, this work is the first to report its dual capacity for pollutant removal and metabolite production in rum distillery stillage. Compared to bacterial systems *Ganoderma* offers advantages in degrading complex organic compounds and a morphology that supports reuse across treatment cycles. As wastewater research increasingly integrates remediation with metabolite recovery, organisms such as *Ganoderma* are an attractive platform.

## 4. Conclusions

Stillage, at 25% concentration without pH adjustment or supplementation, provided a nutrient profile favorable for biomass accumulation and metabolite synthesis. There was a notable decrease in soluble organic carbon and phenolic compounds in the stillage medium. Biomass yields, β-glucan, and lipid content were on par or better in stillage, while protein content, extractable phenolics and antioxidant capacity were relatively close to those produced in a nutrient-rich medium. Stillage is effectively pre-sterilized, representing a significant saving in operational expenditure for media preparation. The mycelial biomass presents an opportunity for valorization. Fungal mycelia are established as components of food products and the presence of β-glucans, proteins, and antioxidants further supports its use in functional foods and nutraceuticals. While requiring optimization for scale-up, the capacity of *Ganoderma* to grow on stillage, reduce environmental pollutants, and yield multiple bioactive products reinforces the broader role of fungi in circular sustainable bioprocesses.

## Figures and Tables

**Table 1:** Macroscopic morphological characteristics of *Ganoderma* isolates G1–G7.

Isolate	Description
G1	Laccate, fan-shaped pileus. Dark brown becoming lighter at margins. Concentric zones on pileus surface. Margin and pore surface are cream white. Pore layer is thin, with thicker context tissue.
G2	Laccate, kidney-shaped pileus. Dark-brown surface with dark-brown margins. Concentric zones on pileus surface. Pore surface darkened due to physical damage (common in *Ganoderma* species). Pore layer thicker than context tissue.
G3	Laccate, kidney-shaped pileus. Dark brown becoming lighter at margins. Concentric zones on pileus surface. Both margin and pore surface are cream white. Context tissue is thicker than pore layer.
G4	Laccate, kidney-shaped pileus. Medium-brown color becoming lighter at margins. Margin and pore layer are cream white. Context tissue thicker than pore layer.
G5	Laccate, kidney-shaped pileus. Medium-brown pileus becoming lighter at margin. Margin and pore surface are cream white. Context tissue thicker than pore layer.
G6	Laccate, fan-shaped pileus. Dark brown with concentric zones on surface. Margin dark. Cream white pore surface, thicker than context tissue.
G7	Laccate, fan-shaped pileus. Medium brown with concentric zones. Margins and pore tissue are cream white. Pore layer thicker than context tissue.

**Table 2:** Chemical characteristics of rum stillage collected on 16/03/2024. Total phenolic content is expressed as gallic acid equivalents (GAE).

Parameter	Value
pH	4.85
Soluble organic carbon	14.7 g·L^−1^
Total nitrogen	0.7 g·L^−1^
Total phenolic content	1.8 g GAE·L^−1^

## Data Availability

The datasets generated during and/or analyzed during the current study are available from the corresponding authors upon reasonable request.

## Supplementary Materials

The following supporting information can be downloaded at: https://www.mdpi.com/article/10.3390/jof11060432/s1, Figure S1:Preliminary assessment of biomass yields (g·L^−1^) of two basidiomycete strains (*Pleurotus eryngii*), and an ascomycete (*Trichoderma reesei*) after 12 days of submerged cultivation in media with increasing concentrations of stillage (0–100%); Tables S1–S6: Pearson correlation coefficients (r) for isolate G1, G2, and G3 cultivated in 25% stillage or nutrient medium at original pH over 12 days
