# Supplementary material for: Conversion of Soluble Compounds in Distillery Wastewater into Fungal Biomass and Metabolites Using Australian Ganoderma Isolates
Source: J Fungi (Basel). 2025 Jun 6;11(6):432. doi: 10.3390/jof11060432 (PMC12194575; doi:10.3390/jof11060432)
Supplement: Supplementary file 1 [file jof-11-00432-s001.zip › jof-3653561-supplementary.pdf]

Supplementary material

Conversion of soluble compounds in distillery wastewater into fungal biomass and metabolites using Australian *Ganoderma* isolates

Aline D.O. Campos [1,2], Hashini J. Wahalathanthrige [2], Shane Russell [4], Mark D. Harrison [1,3], and P. James Strong [1,2]*

[1]Centre for Agriculture and the Bioeconomy, Queensland University of Technology, Brisbane, Australia; aline.deoliveiracampos@hdr.qut.edu.au (A.C.); md.harrison@qut.edu.au (M.H.)

[2]School of Biology and Environmental Science, Queensland University of Technology, Brisbane, Australia

[3]School of Mechanical, Medical, and Process Engineering, Queensland University of Technology, Brisbane, Australia

[4]Central Analytical Research Facility, Queensland University of Technology, Brisbane, Queensland, Australia; sc.russell@qut.edu.au (S.R.)

*Correspondence: pjstrong@gmail.com (J.S.)

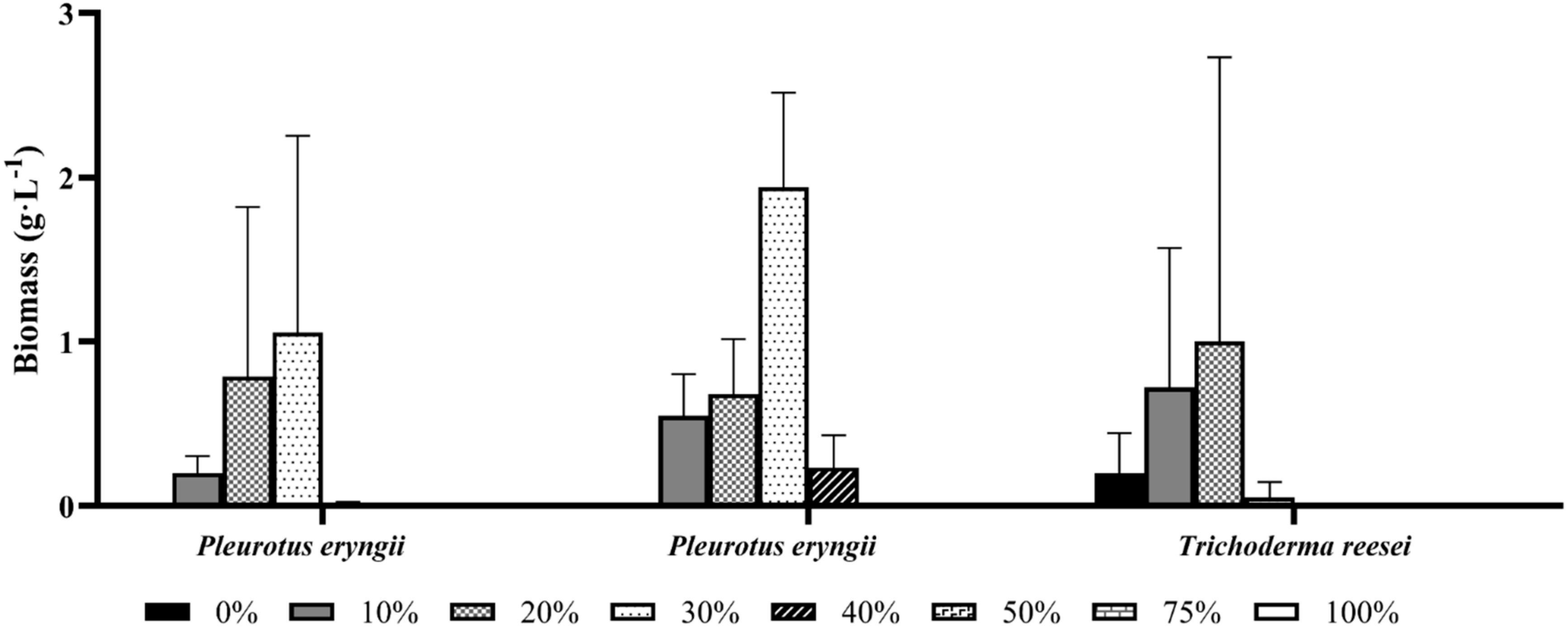

Figure S1. Preliminary assessment of biomass yields (g·L^{-1}) of two basidiomycete strains (*Pleurotus eryngii*), and an ascomycete (*Trichoderma reesei*) after 12 days of submerged cultivation in media with increasing concentrations of stillage (0-100%). Fungi were cultured at 25 °C in 24-well flasks shaking at 150 rpm. Biomass was collected in a sieve, rinsed with distilled water and oven-dried. Error bars represent standard deviations from triplicate cultures.

Table S1. Pearson correlation coefficients (r) for isolate G1 cultivated in 25% stillage at original pH over 12 days. Pairwise Pearson correlation coefficients (n = 4) between biomass, bioremediation indicators (soluble organic carbon, SOC; total phenolic content, TP; total nitrogen, TN), and metabolite production (β-glucan, soluble proteins (SP), crude proteins (CP), phenolics, lipids, 2,2'-azino-bis(3-ethylbenzothiazoline-6-sulphonic acid (ABTS), and Ferric Reducing Antioxidant Power (FRAP)). Asterisks indicate significance levels (*p < 0.05, **p < 0.01, ***p < 0.001, ****p < 0.0001).

	**Biomass**	**TOC**	**TP**	**TN**	**β-glucan**	**Lipids**	**ABTS**	**CP**	**SP**	**Phenolics**	**FRAP**
**Biomass**	1.00	**0.97***	**0.99***	0.87	0.92	0.58	0.29	0.94	-0.67	-0.50	0.59
**TOC**		1.00	**0.96***	0.95	0.89	0.51	0.11	0.87	-0.82	-0.58	0.38
**TP**			1.00	0.83	0.85	0.69	0.37	**0.97***	-0.65	-0.39	0.61
**TN**				1.00	0.91	0.21	-0.20	0.67	-0.94	-0.80	0.13
**β-glucan**					1.00	0.23	-0.01	0.75	-0.73	-0.76	0.42
**Lipids**						1.00	0.85	0.81	-0.03	0.40	0.74
**ABTS**							1.00	0.59	0.45	0.66	0.88
**CP**								1.00	-0.44	-0.17	0.77
**SP**									1.00	0.83	0.20
**Phenolics**										1.00	0.25
**FRAP**											1.00

Table S2. Pearson correlation coefficients (r) for isolate G1 cultivated in nutrient medium (yeast extract, malt extract, peptone and glucose) over 12 days. Correlation matrix (n = 4) showing relationships among biomass and metabolite production (β-glucan, proteins, phenolics, lipids, 2,2'-azino-bis(3-ethylbenzothiazoline-6-sulphonic acid (ABTS), and Ferric Reducing Antioxidant Power (FRAP)). Asterisks indicate significance levels (*p < 0.05, **p < 0.01, ***p < 0.001, ****p < 0.0001).

	Biomass	β-glucan	Lipids	ABTS	CP	SP	Phenolics	FRAP
**Biomass**	1	0.87	0.89	0.74	0.93	**0.98***	0.82	0.93
**β-glucan**		1	**0.97***	0.95	**0.98***	**0.95***	0.93	0.63
**Lipids**			1	0.85	0.94	0.94	0.82	0.68
**ABTS**				1	0.94	0.86	**0.98***	0.47
**CP**					1	**0.98***	**0.96***	0.74
**SP**						1	0.91	0.84
**Phenolics**							1	0.61
**FRAP**								1

Table S3. Pearson correlation coefficients (r) for isolate G2 cultivated in 25% stillage at original pH over 12 days. Pairwise Pearson correlation coefficients (n = 4) between biomass, bioremediation indicators (TOC, TP, TN reduction), and metabolite levels (β-glucan, proteins, phenolics, lipids, ABTS, FRAP). Asterisks indicate significance levels (*p < 0.05, **p < 0.01, ***p < 0.001, ****p < 0.0001).

	Biomass	TOC	TP	TN	β-glucan	Lipids	ABTS	CP	SP	Phenolics	FRAP
**Biomass**	1.00	0.82	0.81	0.71	0.83	0.14	-0.29	-0.47	-0.59	-0.65	0.92
**TOC**		1.00	**1.00*** **	**0.98***	0.89	0.68	0.30	0.11	-0.03	-0.66	0.92
**TP**			1.00	**0.99***	0.88	0.68	0.30	0.12	-0.02	-0.64	0.92
**TN**				1.00	0.81	0.80	0.42	0.28	0.12	-0.56	0.84
**β-glucan**					1.00	0.40	0.22	-0.17	-0.14	-0.93	**0.98***
**Lipids**						1.00	0.81	0.80	0.66	-0.17	0.37
**ABTS**							1.00	0.85	0.94	-0.21	0.08
**CP**								1.00	0.92	0.30	-0.24
**SP**									1.00	0.12	-0.27
**Phenolics**										1.00	-0.85
**FRAP**											1.00

Table S4. Pearson correlation coefficients (r) for isolate G2 cultivated in nutrient medium (yeast extract, malt extract, peptone and glucose) over 12 days. Correlation matrix (n = 4) showing relationships among biomass production, antioxidant capacity (ABTS, FRAP), and metabolite production. Asterisks indicate significance levels (*p < 0.05, **p < 0.01, ***p < 0.001, ****p < 0.0001).

	Biomass	β-glucan	Lipids	ABTS	CP	SP	Phenolics	FRAP
**Biomass**	1.00	0.90	0.80	0.78	0.72	0.89	0.94	**0.99***
**β-glucan**		1.00	0.62	0.44	0.36	0.82	0.78	0.95
**Lipids**			1.00	0.88	0.86	0.95	**0.96***	0.72
**ABTS**				1.00	**1.00****	0.75	0.89	0.67
**CP**					1.00	0.71	0.85	0.60
**SP**						1.00	**0.96***	0.85
**Phenolics**							1.00	0.88
**FRAP**								1.00

Table S5. Pearson correlation coefficients (r) for isolate G3 cultivated in 25% stillage at original pH over 12 days. Pairwise Pearson correlation coefficients (n = 4) between biomass, bioremediation indicators (TOC, TP, TN reduction), and metabolite levels (β-glucan, proteins, phenolics, lipids, ABTS, FRAP). Asterisks indicate significance levels (*p < 0.05, **p < 0.01, ***p < 0.001, ****p < 0.0001).

	Biomass	TOC	TP	TN	β-glucan	Lipids	ABTS	CP	SP	Phenolics	FRAP
**Biomass**	1.00	**1.00****	**0.98***	**0.99****	0.73	0.36	-0.57	0.20	-0.19	**-0.96***	0.77
**TOC**		1.00	**0.98***	**0.99****	0.76	0.30	-0.61	0.16	-0.24	**-0.98***	0.81
**TP**			1.00	**0.95***	0.70	0.17	-0.73	-0.01	-0.39	**-1.00****	0.76
**TN**				1.00	0.81	0.37	-0.52	0.26	-0.15	-0.95	0.84
**β-glucan**					1.00	0.05	-0.41	0.15	-0.24	-0.74	**1.00****
**Lipids**						1.00	0.51	0.93	0.83	-0.10	0.02
**ABTS**							1.00	0.69	0.90	0.76	-0.50
**CP**								1.00	0.91	0.06	0.09
**SP**									1.00	0.44	-0.31
**Phenolics**										1.00	-0.80
**FRAP**											1.00

Table S6. Pearson correlation coefficients (r) for isolate G3 cultivated in nutrient medium (yeast extract, malt extract, peptone and glucose) over 12 days. Correlation matrix (n = 4) showing relationships among biomass production, antioxidant capacity (ABTS, FRAP), and metabolite production. Asterisks indicate significance levels (*$p < 0.05$, **$p < 0.01$, ***$p < 0.001$, ****$p < 0.0001$).

	Biomass	β-glucan	Lipids	ABTS	CP	SP	Phenolics	FRAP
**Biomass**	1.00	**0.97***	0.74	0.57	0.45	0.82	0.74	**0.97***
**β-glucan**		1.00	0.85	0.74	0.60	0.93	0.88	0.88
**Lipids**			1.00	**0.95***	0.93	**0.97***	0.83	0.62
**ABTS**				1.00	**0.96***	0.93	0.87	0.39
**CP**					1.00	0.84	0.69	0.31
**SP**						1.00	0.94	0.68
**Phenolics**							1.00	0.55
**FRAP**								1.00
